# Clinical examination of the macula

**Published:** 2025-01-31

**Authors:** Andrew Blaikie

**Affiliations:** 1 Consultant Ophthalmologist: NHS Fife, St Andrews, Scotland; 2 Senior Lecturer University of St Andrews, St Andrews, Scotland; 3 Honorary Associate Professor: LSHTM, International Centre for Eye Health, London, UK.

**The macula needs to be assessed in any patient presenting with loss of vision, particularly if there is a history of visual distortion.**

**Figure:**
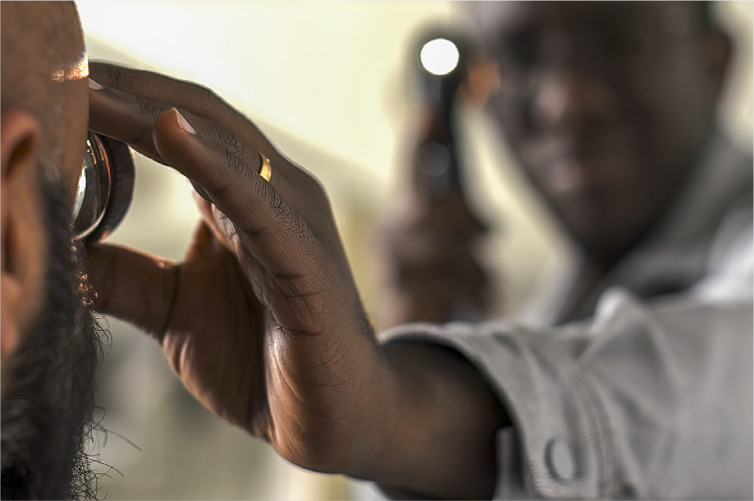
Examining the macula monocularly and indirectly using a direct ophthalmoscope and condensing lens. UK

© ANDREW BLAIKIE, UNIVERSITY OF ST ANDREWS CC BY-NC-SA 4.0

There are three commonly used ‘hands-on’ tools that can be used to examine the macula: the direct ophthalmoscope, slit lamp, and binocular indirect ophthalmoscope. Each of these have different strengths and weaknesses, and provide different fields of view. As with any practical skill, the best results will be achieved with regular practice and mentorship.

## Before you start

There are a number of assessment steps, before looking at the macula, that will help work out what is wrong with the patient. These steps are common to whatever tool is being used.

### Visual acuity

Assess near and distance acuity, including with a pinhole, to exclude refractive error and presbyopia.

### Distortion

This can be detected using an Amsler chart – a 10 cm by 10 cm square grid of 5 mm squares with a fixation spot in the centre. Disturbance in the appearance of the grid can suggest macular disease. However, looking at any object with straight lines, such as a window frame or doorway, is an effective means of detecting distorted vision.

### Pupils

Perform the direct and swinging pupillary light tests to look for an afferent and relative afferent pupillary defect (RAPD), respectively. An afferent defect is present when the pupil either fails to constrict or does not constrict as briskly as expected on direct illumination. A relative afferent pupillary defect is present when, during the swinging light test’, the pupil is seen to paradoxically dilate on illumination. If this sign is present, then the patient is likely to have asymmetrical optic nerve disease or a significant widespread retinal disorder, such as retinal detachment or a vascular occlusion. An afferent or RAPD is considered uncommon in a purely macular condition, although it can occur. Read more at www.cehjournal.org/articles/412

## Tips for finding the macula

- Examine the patient in a dim room.
- Dilate the pupils. Even very experienced eye care practitioners will struggle to examine the macula through an undilated pupil. This is because the pupil will constrict as soon as light falls on the macula, limiting your view.
- Start with a low light and gradually increase the brightness, so that you can balance the comfort of the patient with your ability to see the detail of the macula.
- In general, to find the macula, first find the optic nerve. Note its contour, colour, and cup. Look temporally, or ask the patient to look at the light. This should bring the macula into view. If not dilated, the pupil will constrict and the view will be lost.
- The macula typically appears slightly darker than the surrounding retina, with a central bright foveal reflex.

### Using a direct ophthalmoscope

When examining the macula, it can be best to start with the patient and the examiner wearing their usual distance refractive correction with the ophthalmoscope set to zero.

Use your right eye to examine the patient's right eye (and your left eye to examine the patient's left eye).

**Figure 1:**
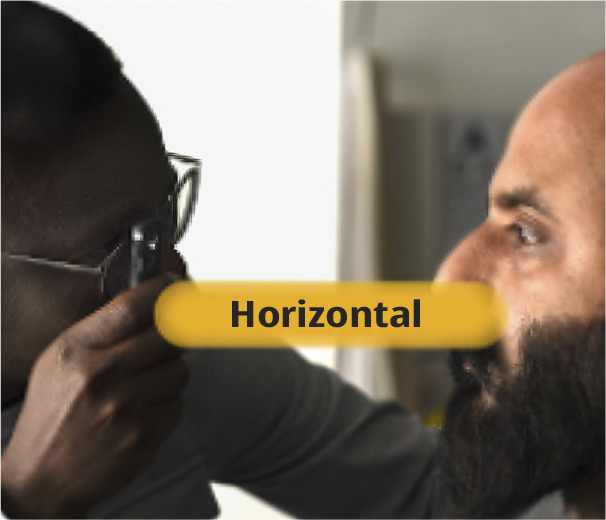
Position yourself so your eye is the same horizontal level as the patient's.

Position your eye at the same horizontal level as that of the patient (Figure 1) and ask the patient to look at a point straight in front of them.

**Figure 2:**
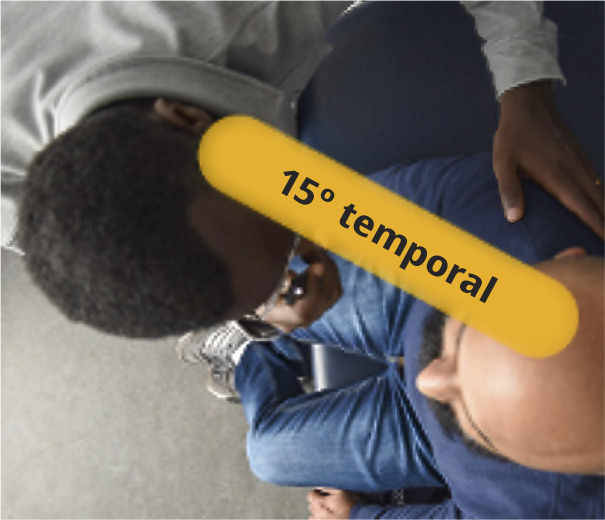
Lean back and 15 degrees temporally to see the fundal reflex.

Stand with your feet close to the patient. Lean back and 15 degrees to the temporal side (Figure 2), until you can see the fundal ‘red’ reflex in both eyes to assess the clarity of the media.

© ANDREW BLAIKIE, UNIVERSITY OF ST ANDREWS CC BY-NC-SA 4.0

**Figure 3:**
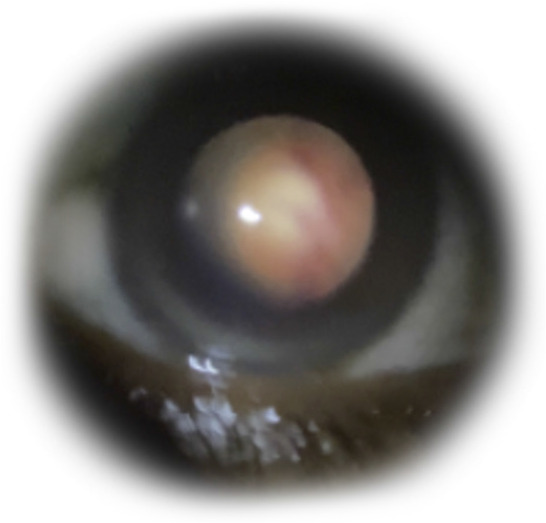
The optic disc.

From there, slowly lean towards the patient, following the fundal reflex on this horizontal 15-degree temporal ‘flight path’ to find the optic disc (Figure 3). At first it will appear a bit blurred, but as you get closer it should become clearer. Use the lenses in the ophthalmoscope if it remains blurred.

© ANDREW BLAIKIE, UNIVERSITY OF ST ANDREWS CC BY-NC-SA 4.0

**Figure 4:**
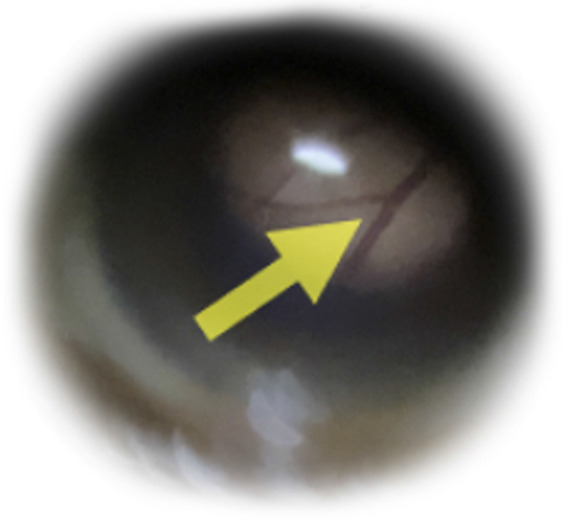
The V shape created where the blood vessels join (see yellow arrow) is like an arrow pointing towards the optic disc.

If you can't find the optic disc, find where the blood vessels join. The natural V shape created where they join is like an arrow pointing towards the disc. Move in this direction to find the disc (Figure 4).

© ANDREW BLAIKIE, UNIVERSITY OF ST ANDREWS CC BY-NC-SA 4.0

**Figure 5:**
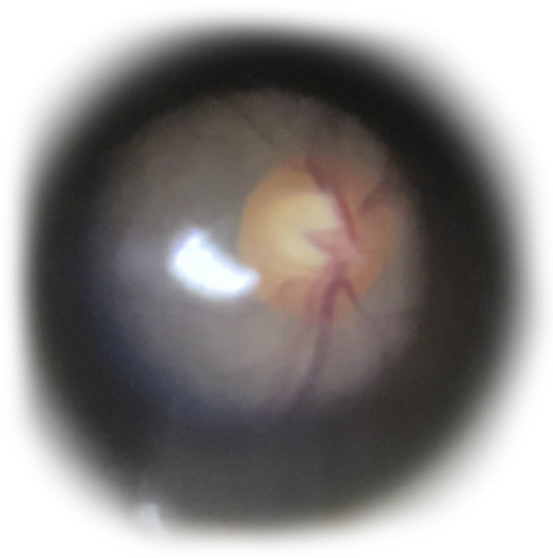
A closer view of the optic disc.

Once you locate the optic disc, move closer to the patient get a clearer view (Figure 5). Comment on the contour, colour, and cup of the optic disc.

© ANDREW BLAIKIE, UNIVERSITY OF ST ANDREWS CC BY-NC-SA 4.0

**Figure 6:**
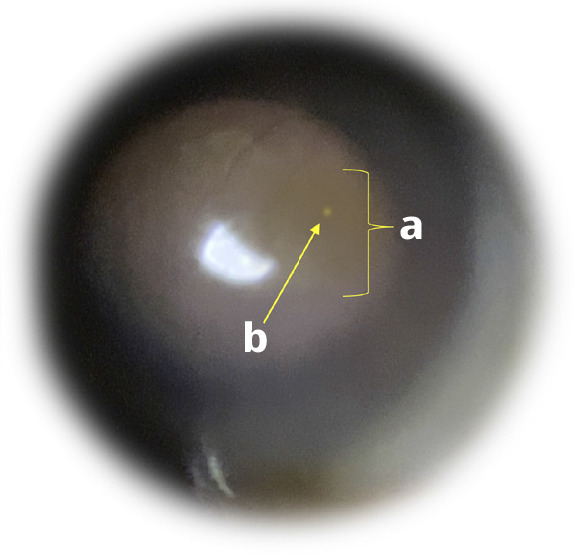
The macula is the darker area **(a)**. Notice the small bright area in the centre – this is the foveal reflex **(b)**.

From the optic disc, move temporally until you can see the darker macula and bright foveal reflex (Figure 6).

## Using a slit lamp

**Figure 7.**
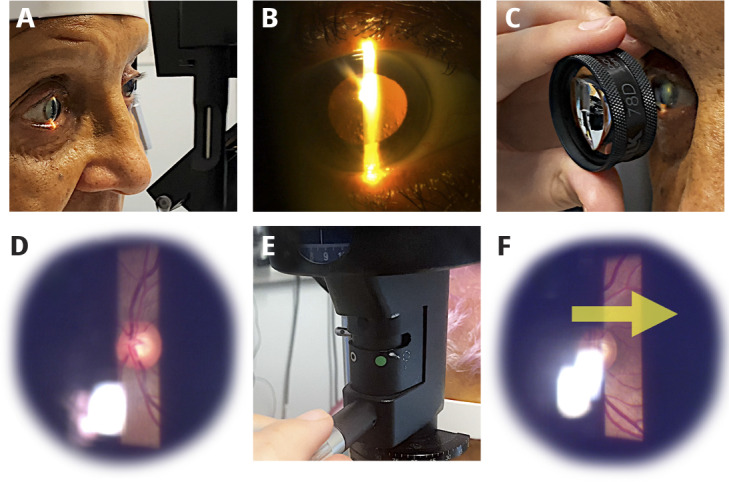
**A:** Forehead against the bar. **B:** Axial illumination; check the reflex. **C:** Brace the lens and hold the upper lid. **D:** Find the disc. **E:** Adjust the light and lens position to optimise the view. **F:** Move temporally to find the macula and fovea, remembering that what is seen is reversed horizontally and vertically.

Position the patient so that their eyes are at the same level as the black mark on the vertical bar and their head is forward, leaning against the forehead strap (Figure 7A).

© ANDREW BLAIKIE, UNIVERSITY OF ST ANDREWS CC BY-NC-SA 4.0

Line up the light source and eye pieces to create axial illumination (Figure 7B). Use your fingers to brace the position of the lens and hold the eyelid up to reduce blinking. Ask the patient to look slightly temporally, towards your ear.

The optic disc should come into view. Adjust the tilt/distance of your lens and the beam brightness, width, and height to optimise the view. Then move temporally to find macula (Figure 7).

## Using a binocular indirect ophthalmoscope

Patients are best examined lying down, but sitting is okay. Make sure the binocular indirect ophthalmoscope feels comfortable and it is aligned with your eyes so you can see your thumb in the centre of each eyepiece with your arm straight out in front of you.

**Figure 8.**
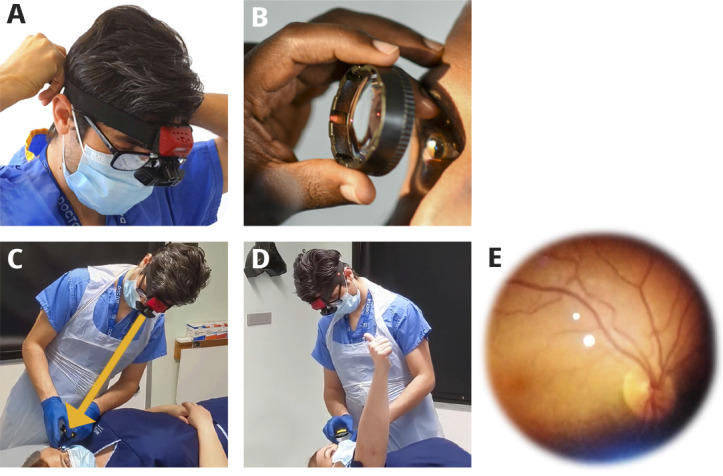
**A:** Ensure the binocular indirect ophthalmoscope fits comfortably. **B:** Use your fingers to brace the lens and hold the upper eyelid. **C:** Line up the eye, lens, and ophthalmoscope as you check the reflex. **D:** Use the patient's thumb as a target. **E:** Typical view of the disc and macula.

Use your fingers to brace the lens at the correct distance and hold the patient's upper eyelid open. Ask the patient to look at their thumb. Move the position of their thumb to move their gaze; this will allow you to examine of all parts of the fundus. Tilting the lens and varying the brightness of the light can improve the view (Figure 8).

© ANDREW BLAIKIE, UNIVERSITY OF ST ANDREWS CC BY-NC-SA 4.0

**Figure 9:**
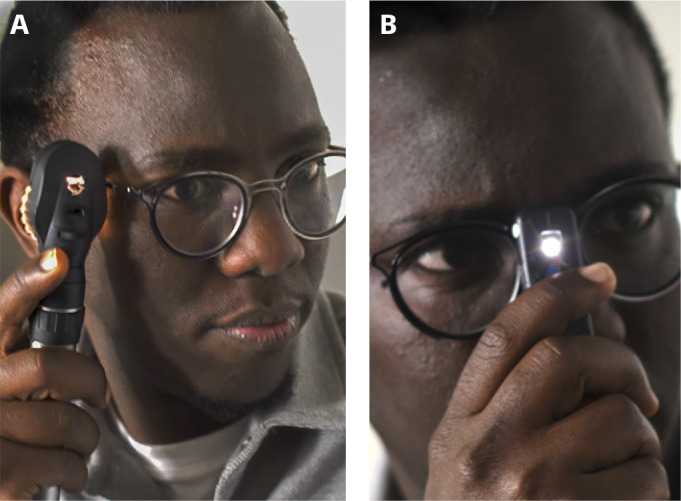
Indirect ophthalmoscopy using the direct ophthalmoscope as a light source. **A:** Monocular view if held to the side **B:** Binocular view is possible if an Arclight is held between the eyes and both eyes are used to view the fundus.

Indirect ophthalmoscopy can also be performed using the light source of a direct ophthalmoscope. Placing it by the side of the eye allows a monocular view, and between the eyes a binocular indirect view. This is most effective through a dilated pupil, and presbyopic examiners will need to wear near correction (Figure 9).

© ANDREW BLAIKIE, UNIVERSITY OF ST ANDREWS CC BY-NC-SA 4.0

**Videos**

For a playlist of videos about examining the macula, visit tinyurl.com/bdz3e92t

## Which tool is the best?

**Figure 10:**
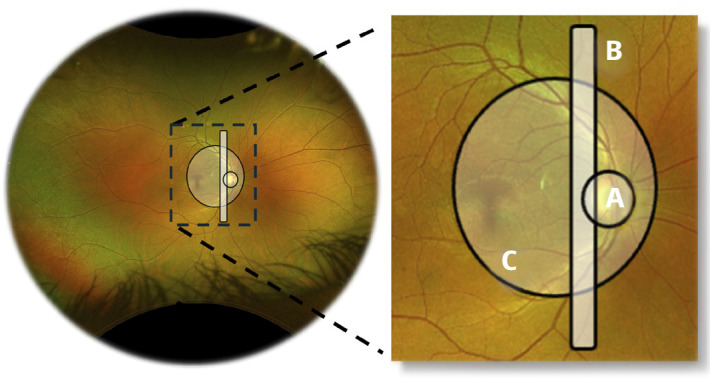
Relative size of fields of view superimposed upon a widefield Optomap **A:** Direct ophthalmoscope, **B:** Slit lamp, **C:** Binocular indirect ophthalmoscope.

Different devices and lenses have different optical properties. In general, the greater the magnification, the smaller the field of view (Table 1). For instance, a direct ophthalmoscope has a high magnification, but a very small, monocular field of view. In Figure 10, this is represented by the circle labelled A. Both the slit lamp (B) and the binocular indirect ophthalmoscope (C) offer less magnification but wider, stereo fields of view.

**Table 1:** Magnification and field of view provided by different tools and lenses. Note: Slit lamp magnifications shown are for a slit lamp at 10x magnification; however, this can be adjusted from 6x to 40x.

Device	Condensing lens power	Magnification	Field of view (degrees)
Direct Ophthalmoscope	N/A	15x	10-15
Slit lamp	60D	11.5x	68–81
	66D	10x	80–96
	78D	9.3x	81–97
	90D	7.6x	74–89
Indirect ophthalmoscope	20D	3x	45–50
	28D	2x	53–58
	30D	1.9x	60–65

The different devices also have a range of different features and functionality (Table 2). The slit lamp offers excellent anterior segment examination but is the least portable and most expensive. The binocular indirect ophthalmoscope is typically the best at offering a useful view through hazy media. Both the slit lamp and indirect ophthalmoscope require additional condensing lenses; because of this, the direct ophthalmoscope is often considered the easiest to use.

**Table 2:** Relative strengths and weaknesses of the direct ophthalmoscope, slit lamp and indirect ophthalmoscope.

	Direct ophthalmoscope	Slit lamp	Indirect ophthalmoscope
Portable	+++	-	++
Cost	$	$$$	$$
Magnification	+++	++	-
Field of view	+	++	+++
Binocularity (3D view)	-	+++	+
Ease of use	++	+	+
Can see through cataract	+	++	+++

The preferred technique for examining the macula is the slit lamp, with a 78D lens. However, all the methods are effective, and your choice will depend on several factors.

As with any practical skill, the best results will be achieved with regular practice and mentorship. For instance, someone who most frequently uses a direct ophthalmoscope will be able to detect disease more accurately than someone who only occasionally uses a slit lamp.

